# Supplementary material for: A Mathematical Radiobiological Model (MRM) to Predict Complex DNA Damage and Cell Survival for Ionizing Particle Radiations of Varying Quality
Source: Molecules. 2021 Feb 5;26(4):840. doi: 10.3390/molecules26040840 (PMC7914858; doi:10.3390/molecules26040840)
Supplement: Supplementary file 1 [file molecules-26-00840-s001.pdf]

molecules

Supplementary information

A Mathematical Radiobiological Model (MRM) to Predict Complex DNA Damage and Cell Survival for Ionizing Particle Radiations of Varying Quality

Spyridon A. Kalospyros [1], Zacharenia Nikitaki [1], Ioanna Kyriakou [2], Michael Kokkoris [1], Dimitris Emfietzoglou [2] and Alexandros G. Georgakilas [1,*]

[1] Physics Department, School of Applied Mathematical and Physical Sciences, National Technical University of Athens (NTUA), 15780 Zografou, Greece; spkals@central.ntua.gr (S.A.K.); znikitaki@mail.ntua.gr (Z.N.); kokkoris@central.ntua.gr (M.K.)

[2] Medical Physics Lab, Department of Medicine, University of Ioannina, 45110 Ioannina, Greece; ikyriak@uoi.gr (I.K.); demfietz@uoi.gr (D.E.)

* Correspondence: alexg@mail.ntua.gr; Tel.: +30-210-772-4453

APPENDIX A

In our MRM methodology, we have used the ion's LET as an input parameter. As referred above, to a first approximation, in the case of ions, the linear energy transfer (LET) can be considered identical to the stopping power S_e. Also, as explained, according to the dielectric formalism [139], through the kinetic energy T of an ion-projectile, one can calculate their total ionization cross sections (TICS) in liquid water. And since the latter represent the number $\frac{dNe}{d\zeta}$ of secondary electrons emitted per unit length ζ from the ion's path, we may combine the values of these 3 quantities (T, S_e and $\frac{dNe}{d\zeta}$) in a table for all the ions that we have used.

In **Table S1** are shown the values of the kinetic energy T (MeV), the stopping power S_e (eV/nm) and the number of secondary electrons (e^-) ejected per nm of the ion's path for all the ions used in this paper (protons, ^{12}C, ^{11}B, ^{14}N, ^{16}O, ^{20}Ne, ^{40}Ar, ^{28}Si and ^{56}Fe).

We have used the data for protons and carbon ions in liquid water from the appendix of ICRU Report 90 [150] and the corresponding data for the rest of the heavy ions (except for ^{56}Fe) from the ICRU Report 73 [151] while the data for ^{56}Fe have been taken from SRIM 2008 [152].

Therefore, knowing the ion's energy, we also know their stopping power, as well as the number $\frac{dNe}{d\zeta}$ of secondary electrons emitted per unit length of an ion's path.

<u>Table S1</u>: The values of the kinetic energy *T* (MeV), the stopping power S_e (eV/nm) and the number of secondary electrons ejected per nm of the ion's path ($\frac{dNe}{d\zeta}$) for all the ions used in this paper (protons,^{12}C,^{11}B, ^{14}N, ^{16}O, ^{20}Ne, ^{40}Ar, ^{28}Si and ^{56}Fe). The columns of *T* and S_e for protons and carbon ions have been taken from ICRU Report 90, [150] the corresponding ones for the ions ^{11}B, ^{14}N, ^{16}O, ^{20}Ne, ^{40}Ar and^{28}Si are from ICRU Report 73 [151] and for ^{56}Fe are from SRIM 2008 [152].

$_{14}$N			$_{20}$Ne			$_{28}$Si		
T (Mev)	Se (eV/nm)	dNe/dζ(e-/nm)	T (Mev)	Se (eV/nm)	dNe/dζ(e-/nm)	T (Mev)	Se (eV/nm)	dNe/dζ(e-/nm)
0.35025	4.481E+02	0.897721971	0.5045	5.172E+02	1.195315893	0.70225	5.893E+02	1.557904905
0.4203	4.974E+02	1.45410518	0.6054	5.785E+02	1.945185836	0.8427	6.570E+02	2.54415951
0.5604	5.848E+02	3.187331614	0.8072	6.917E+02	4.298818497	1.1236	7.852E+02	5.657411074
0.7005	6.603E+02	5.39011584	1.009	7.932E+02	7.321670045	1.4045	9.055E+02	9.687663858
0.8406	7.261E+02	7.186697061	1.2108	8.847E+02	9.824331767	1.6854	1.018E+03	13.06197324
0.9807	7.838E+02	8.459957705	1.4126	9.674E+02	11.63203491	1.9663	1.123E+03	15.53368986
1.1208	8.343E+02	9.373608998	1.6144	1.042E+03	12.9572118	2.2472	1.221E+03	17.37392276
1.2609	8.781E+02	10.05030914	1.8162	1.110E+03	13.961769	2.5281	1.311E+03	18.79208334
1.401	9.161E+02	10.5649952	2.018	1.172E+03	14.74523947	2.809	1.396E+03	19.91748748
2.1015	1.037E+03	11.88598806	3.027	1.395E+03	16.92251425	4.2135	1.737E+03	23.21165954
2.802	1.087E+03	12.30176922	4.036	1.518E+03	17.80029769	5.618	1.962E+03	24.72452044
3.5025	1.105E+03	12.37608717	5.045	1.582E+03	18.15689348	7.0225	2.106E+03	25.49386241
4.203	1.106E+03	12.29377355	6.054	1.614E+03	18.25621058	8.427	2.197E+03	25.87943444
5.604	1.088E+03	11.93148027	8.072	1.630E+03	18.08889349	11.236	2.286E+03	26.06804263
7.005	1.059E+03	11.47896984	10.09	1.618E+03	17.70791779	14.045	2.316E+03	25.87844934
8.406	1.028E+03	11.01457262	12.108	1.595E+03	17.2487813	16.854	2.317E+03	25.51761475
9.807	9.954E+02	10.56546146	14.126	1.566E+03	16.76628408	19.663	2.304E+03	25.0756836
11.208	9.641E+02	10.14109354	16.144	1.535E+03	16.28494533	22.472	2.282E+03	24.59697258
12.609	9.338E+02	9.744010397	18.162	1.503E+03	15.81619283	25.281	2.256E+03	24.10495343
14.01	9.050E+02	9.373904454	20.18	1.471E+03	15.36525813	28.09	2.228E+03	23.61262797
21.015	7.807E+02	7.867907638	30.27	1.321E+03	13.40954696	42.135	2.072E+03	21.31588998
28.02	6.845E+02	6.779464972	40.36	1.196E+03	11.88549027	56.18	1.924E+03	19.38097612
35.025	6.085E+02	5.958949755	50.45	1.089E+03	10.67610034	70.225	1.791E+03	17.76595697
42.03	5.473E+02	5.318336723	60.54	9.990E+02	9.694326002	84.27	1.675E+03	16.40421513
56.04	4.551E+02	4.382195149	80.72	8.567E+02	8.196505818	112.36	1.480E+03	14.23712986
70.05	3.892E+02	3.730624736	100.9	7.488E+02	7.106171877	140.45	1.323E+03	12.58784461
84.06	3.401E+02	3.25079386	121.08	6.647E+02	6.275960565	168.54	1.197E+03	11.2884363
98.07	3.023E+02	2.882637577	141.26	5.974E+02	5.622254695	196.63	1.092E+03	10.23687713
112.08	2.722E+02	2.591212172	161.44	5.425E+02	5.0939491	224.72	1.004E+03	9.367813973
126.09	2.477E+02	2.354767261	181.62	4.969E+02	4.658113194	252.81	9.285E+02	8.637001489
140.1	2.275E+02	2.159062421	201.8	4.586E+02	4.292315856	280.9	8.639E+02	8.01367816
210.15	1.633E+02	1.533563112	302.7	3.325E+02	3.092445697	421.35	6.417E+02	5.900987849
280.2	1.288E+02	1.196236046	403.6	2.630E+02	2.426766821	561.8	5.125E+02	4.681362748
350.25	1.074E+02	0.984670073	504.5	2.191E+02	2.003057123	702.25	4.285E+02	3.88714775
420.3	9.255E+01	0.839103924	605.4	1.888E+02	1.709306117	842.7	3.698E+02	3.328674125
560.4	7.339E+01	0.651050721	807.2	1.496E+02	1.327806007	1123.6	2.932E+02	2.594789753
700.5	6.144E+01	0.53424304	1009	1.252E+02	1.089976063	1404.5	2.453E+02	2.133307291
840.6	5.322E+01	0.454309446	1210.8	1.085E+02	0.927028912	1685.4	2.125E+02	1.815685811
980.7	4.722E+01	0.396004851	1412.6	9.627E+01	0.808106285	1966.3	1.885E+02	1.583293696
1120.8	4.261E+01	0.3515073	1614.4	8.692E+01	0.717311845	2247.2	1.703E+02	1.405674712
1260.9	3.898E+01	0.316373074	1816.2	7.953E+01	0.645629984	2528.1	1.558E+02	1.265343637
1401	3.604E+01	0.287890604	2018	7.353E+01	0.587519284	2809	1.441E+02	1.15143851
2101.5	2.695E+01	0.200026704	3027	5.503E+01	0.408216681	4213.5	1.080E+02	0.800056962
2802	2.225E+01	0.154323805	4036	4.546E+01	0.314948234	5618	8.924E+01	0.617286842
3502.5	1.938E+01	0.12613055	5045	3.961E+01	0.257411148	7022.5	7.780E+01	0.504531686
4203	1.746E+01	0.106929321	6054	3.569E+01	0.218220773	8427	7.012E+01	0.427730791
5604	1.504E+01	0.082350944	8072	3.077E+01	0.168061087	11236	6.049E+01	0.329410927
7005	1.362E+01	0.067217128	10090	2.786E+01	0.137180088	14045	5.479E+01	0.268872129
8406	1.269E+01	0.056923854	12108	2.597E+01	0.116173839	16854	5.109E+01	0.227697469
9807	1.206E+01	0.049450969	14126	2.467E+01	0.100924184	19663	4.852E+01	0.197804778
11208	1.160E+01	0.043769477	16144	2.373E+01	0.089329041	22472	4.669E+01	0.175077467
12609	1.126E+01	0.039298267	18162	2.303E+01	0.080202669	25281	4.533E+01	0.157192434
14010	1.100E+01	0.035684273	20180	2.250E+01	0.072826219	28090	4.429E+01	0.142735249

^{12}C			protons		
T (Mev)	Se (eV/nm)	dNe/dζ(e-/nm)	T (Mev)	Se (eV/nm)	dNe/dζ(e-/nm)
0.0010	1.177E+02	2.742E-09	0.0010	1.765E+01	1.081E-05
0.0015	1.251E+02	1.383E-08	0.0015	1.980E+01	4.614E-05
0.0020	1.294E+02	4.323E-08	0.0020	2.180E+01	1.243E-04
0.0030	1.339E+02	2.130E-07	0.0030	2.539E+01	4.715E-04
0.0040	1.361E+02	6.536E-07	0.0040	2.858E+01	1.156E-03
0.0050	1.371E+02	1.549E-06	0.0050	3.147E+01	2.249E-03
0.0060	1.378E+02	3.117E-06	0.0060	3.413E+01	3.797E-03
0.0080	1.386E+02	9.288E-06	0.0080	3.892E+01	8.366E-03
0.0100	1.392E+02	2.142E-05	0.0100	4.320E+01	1.499E-02
0.0150	1.410E+02	9.473E-05	0.0150	5.100E+01	4.075E-02
0.0200	1.434E+02	2.641E-04	0.0200	5.721E+01	8.015E-02
0.0300	1.493E+02	1.065E-03	0.0300	6.658E+01	2.075E-01
0.0400	1.561E+02	2.749E-03	0.0400	7.309E+01	4.168E-01
0.0500	1.631E+02	5.593E-03	0.0500	7.753E+01	6.549E-01
0.0600	1.702E+02	9.822E-03	0.0600	8.035E+01	8.187E-01
0.0800	1.843E+02	2.313E-02	0.0800	8.244E+01	9.572E-01
0.1000	1.984E+02	4.375E-02	0.1000	8.145E+01	9.849E-01
0.1500	2.334E+02	1.320E-01	0.1500	7.357E+01	9.270E-01
0.2000	2.668E+02	2.794E-01	0.2000	6.581E+01	8.385E-01
0.3000	3.271E+02	7.915E-01	0.3000	5.430E+01	6.883E-01
0.4000	3.806E+02	1.700E+00	0.4000	4.639E+01	5.804E-01
0.5000	4.289E+02	3.107E+00	0.5000	4.061E+01	5.016E-01
0.6000	4.730E+02	4.705E+00	0.6000	3.620E+01	4.420E-01
0.8000	5.504E+02	7.019E+00	0.8000	2.994E+01	3.582E-01
1.0000	6.162E+02	8.316E+00	1.0000	2.572E+01	3.022E-01
1.5000	7.391E+02	9.772E+00	1.5000	1.932E+01	2.194E-01
2.0000	8.156E+02	1.027E+01	2.0000	1.567E+01	1.737E-01
3.0000	8.797E+02	1.040E+01	3.0000	1.159E+01	1.242E-01
4.0000	8.710E+02	1.014E+01	4.0000	9.306E+00	9.760E-02
5.0000	8.370E+02	9.788E+00	5.0000	7.831E+00	8.083E-02
6.0000	8.026E+02	9.410E+00	6.0000	6.791E+00	6.923E-02
8.0000	7.418E+02	8.683E+00	8.0000	5.409E+00	5.413E-02
10.0000	6.920E+02	8.035E+00	10.0000	4.526E+00	4.467E-02
15.0000	5.974E+02	6.754E+00	15.0000	3.264E+00	3.144E-02
20.0000	5.278E+02	5.822E+00	20.0000	2.586E+00	2.446E-02
30.0000	4.291E+02	4.569E+00	30.0000	1.861E+00	1.713E-02
40.0000	3.623E+02	3.766E+00	40.0000	1.476E+00	1.329E-02
50.0000	3.130E+02	3.207E+00	50.0000	1.236E+00	1.090E-02
60.0000	2.746E+02	2.795E+00	60.0000	1.070E+00	9.272E-03
80.0000	2.204E+02	2.228E+00	80.0000	8.563E-01	7.174E-03
100.0000	1.852E+02	1.857E+00	100.0000	7.236E-01	5.875E-03
150.0000	1.344E+02	1.320E+00	150.0000	5.409E-01	4.082E-03
200.0000	1.068E+02	1.031E+00	200.0000	4.461E-01	3.149E-03
300.0000	7.706E+01	7.237E-01	300.0000	3.503E-01	2.182E-03
400.0000	6.115E+01	5.619E-01	400.0000	3.014E-01	1.681E-03
500.0000	5.116E+01	4.614E-01	500.0000	2.725E-01	1.372E-03
600.0000	4.427E+01	3.925E-01	600.0000	2.545E-01	1.162E-03
800.0000	3.535E+01	3.039E-01	800.0000	2.315E-01	8.933E-04
1000.0000	2.979E+01	2.490E-01	1000.0000	2.196E-01	7.283E-04
1500.0000	2.210E+01	1.731E-01	1500.0000	2.066E-01	5.020E-04
2000.0000	1.810E+01	1.337E-01	2000.0000	2.016E-01	3.853E-04
3000.0000	1.399E+01	9.267E-02	3000.0000	1.996E-01	2.651E-04
4000.0000	1.191E+01	7.140E-02	4000.0000	2.006E-01	2.032E-04
5000.0000	1.066E+01	5.830E-02	5000.0000	2.026E-01	1.653E-04
6000.0000	9.842E+00	4.938E-02	6000.0000	2.046E-01	1.396E-04
8000.0000	8.851E+00	3.799E-02	8000.0000	2.086E-01	1.069E-04
10 000.0000	8.295E+00	3.098E-02	10 000.0000	2.126E-01	8.689E-05

^{56}Fe								
**T (Mev)**	**Se (eV/nm)**	**dNe/dζ(e-/nm)**	**T (Mev)**	**Se (eV/nm)**	**dNe/dζ(e-/nm)**	**T (Mev)**	**Se (eV/nm)**	**dNe/dζ(e-/nm)**
0.01	3.96E+01	1.54E-07	1.6	5.91E+02	3.53E+00	275	3.35E+03	3.24E+01
0.011	4.15E+01	2.25E-07	1.7	6.18E+02	4.18E+00	300	3.25E+03	3.12E+01
0.012	4.33E+01	3.16E-07	1.8	6.45E+02	4.90E+00	325	3.16E+03	3.02E+01
0.013	4.51E+01	4.33E-07	2	7.00E+02	6.60E+00	350	3.07E+03	2.92E+01
0.014	4.68E+01	5.78E-07	2.25	7.66E+02	9.18E+00	375	2.99E+03	2.83E+01
0.015	4.85E+01	7.57E-07	2.5	8.34E+02	1.21E+01	400	2.91E+03	2.74E+01
0.016	5.01E+01	9.74E-07	2.75	9.02E+02	1.50E+01	450	2.76E+03	2.58E+01
0.017	5.16E+01	1.23E-06	3	9.71E+02	1.78E+01	500	2.62E+03	2.45E+01
0.018	5.31E+01	1.54E-06	3.25	1.04E+03	2.02E+01	550	2.50E+03	2.32E+01
0.02	5.60E+01	2.32E-06	3.5	1.11E+03	2.23E+01	600	2.39E+03	2.21E+01
0.0225	5.94E+01	3.66E-06	3.75	1.18E+03	2.42E+01	650	2.28E+03	2.11E+01
0.025	6.26E+01	5.49E-06	4	1.26E+03	2.58E+01	700	2.17E+03	2.02E+01
0.0275	6.57E+01	7.91E-06	4.5	1.40E+03	2.85E+01	800	1.99E+03	1.85E+01
0.03	6.86E+01	1.10E-05	5	1.55E+03	3.08E+01	900	1.83E+03	1.72E+01
0.0325	7.14E+01	1.50E-05	5.5	1.70E+03	3.26E+01	1000	1.69E+03	1.60E+01
0.035	7.41E+01	1.98E-05	6	1.84E+03	3.41E+01	1100	1.57E+03	1.50E+01
0.0375	7.66E+01	2.57E-05	6.5	1.99E+03	3.55E+01	1200	1.47E+03	1.41E+01
0.04	7.91E+01	3.28E-05	7	2.13E+03	3.66E+01	1300	1.38E+03	1.33E+01
0.045	8.39E+01	5.11E-05	8	2.40E+03	3.85E+01	1400	1.31E+03	1.25E+01
0.05	8.85E+01	7.56E-05	9	2.65E+03	4.01E+01	1500	1.25E+03	1.19E+01
0.055	9.28E+01	1.08E-04	10	2.88E+03	4.13E+01	1600	1.21E+03	1.13E+01
0.06	9.69E+01	1.48E-04	11	3.09E+03	4.23E+01	1700	1.17E+03	1.08E+01
0.065	1.01E+02	1.99E-04	12	3.28E+03	4.32E+01	1800	1.12E+03	1.03E+01
0.07	1.05E+02	2.60E-04	13	3.45E+03	4.39E+01	2000	1.04E+03	9.51E+00
0.08	1.12E+02	4.21E-04	14	3.60E+03	4.45E+01	2250	9.49E+02	8.64E+00
0.09	1.19E+02	6.41E-04	15	3.73E+03	4.50E+01	2500	8.79E+02	7.93E+00
0.1	1.25E+02	9.29E-04	16	3.85E+03	4.55E+01	2750	8.19E+02	7.32E+00
0.11	1.32E+02	1.30E-03	17	3.96E+03	4.59E+01	3000	7.68E+02	6.80E+00
0.12	1.45E+02	1.75E-03	18	4.05E+03	4.62E+01	3250	7.25E+02	6.35E+00
0.13	1.57E+02	2.31E-03	20	4.20E+03	4.68E+01	3500	6.87E+02	5.96E+00
0.14	1.65E+02	2.97E-03	22.5	4.35E+03	4.73E+01	3750	6.53E+02	5.62E+00
0.15	1.71E+02	3.74E-03	25	4.45E+03	4.76E+01	4000	6.22E+02	5.31E+00
0.16	1.75E+02	4.64E-03	27.5	4.53E+03	4.79E+01	4500	5.69E+02	4.79E+00
0.17	1.79E+02	5.68E-03	30	4.59E+03	4.80E+01	5000	5.26E+02	4.37E+00
0.18	1.81E+02	6.85E-03	32.5	4.64E+03	4.81E+01	5500	4.90E+02	4.01E+00
0.2	1.85E+02	9.65E-03	35	4.67E+03	4.81E+01	6000	4.59E+02	3.72E+00
0.225	1.89E+02	1.41E-02	37.5	4.69E+03	4.81E+01	6500	4.33E+02	3.46E+00
0.25	1.93E+02	1.96E-02	40	4.71E+03	4.80E+01	7000	4.11E+02	3.24E+00
0.275	1.97E+02	2.64E-02	45	4.72E+03	4.78E+01	8000	3.74E+02	2.87E+00
0.3	2.02E+02	3.45E-02	50	4.72E+03	4.76E+01	9000	3.45E+02	2.59E+00
0.325	2.08E+02	4.40E-02	55	4.70E+03	4.73E+01	10000	3.21E+02	2.35E+00
0.35	2.14E+02	5.49E-02	60	4.68E+03	4.69E+01	11000	3.02E+02	2.16E+00
0.375	2.20E+02	6.74E-02	65	4.65E+03	4.65E+01	12000	2.85E+02	2.00E+00
0.4	2.27E+02	8.14E-02	70	4.61E+03	4.61E+01	13000	2.71E+02	1.86E+00
0.45	2.42E+02	1.15E-01	80	4.53E+03	4.53E+01	14000	2.59E+02	1.74E+00
0.5	2.56E+02	1.55E-01	90	4.44E+03	4.44E+01	15000	2.50E+02	1.63E+00
0.55	2.73E+02	2.02E-01	100	4.36E+03	4.36E+01	16000	2.41E+02	1.54E+00
0.6	2.89E+02	2.57E-01	110	4.27E+03	4.28E+01	17000	2.33E+02	1.46E+00
0.65	3.05E+02	3.20E-01	120	4.25E+03	4.20E+01	18000	2.26E+02	1.38E+00
0.7	3.22E+02	3.92E-01	130	4.22E+03	4.12E+01	20000	2.13E+02	1.26E+00
0.8	3.54E+02	5.60E-01	140	4.14E+03	4.04E+01	22500	2.01E+02	1.13E+00
0.9	3.86E+02	7.66E-01	150	4.06E+03	3.97E+01	25000	1.92E+02	1.03E+00
1	4.17E+02	1.01E+00	160	3.98E+03	3.90E+01	27500	1.84E+02	9.40E-01
1.1	4.48E+02	1.30E+00	170	3.91E+03	3.83E+01	30000	1.77E+02	8.69E-01
1.2	4.77E+02	1.64E+00	180	3.84E+03	3.77E+01	32500	1.72E+02	8.08E-01
1.3	5.06E+02	2.02E+00	200	3.72E+03	3.64E+01	35000	1.67E+02	7.55E-01
1.4	5.35E+02	2.46E+00	225	3.58E+03	3.49E+01	37500	1.64E+02	7.09E-01
1.5	5.63E+02	2.97E+00	250	3.46E+03	3.36E+01	40000	1.60E+02	6.68E-01

References

139. Lindhard, J. On the properties of a gas of charged particles. *Dan. Vid. Selsk Mat.-Fys. Medd.* **1954**, *28*, 8.

150. International Commission on Radiation Units and Measurements. Key Data For Ionizing - Radiation Dosimetry: Measurement Standards And Applications (Report 90). *J Int Comm Radiat Units Meas* **2014**, *14*, 79-80.

151. Bimbot, R.; Geissel, H.; Paul, H.; Shinner, A.; Sigmund, P. Stopping of ions heavier than helium ICRU Report 73. Oxford: Oxford University Press: 2005.

152. Zeigler, J.; Ziegler, M.; Biersack, J. SRIM 2008. 04 software package. 2008. Availabe online: http://www.srim.org (accessed on 9-9-20).

© 2021 by the authors. Licensee MDPI, Basel, Switzerland. This article is an open access article distributed under the terms and conditions of the Creative Com-mons Attribution (CC BY) license (http://creativecommons.org/licenses/by/4.0/).
